# Long-term changes in kelp forests in an inner basin of the Salish Sea

**DOI:** 10.1371/journal.pone.0229703

**Published:** 2021-02-17

**Authors:** Helen D. Berry, Thomas F. Mumford, Bart Christiaen, Pete Dowty, Max Calloway, Lisa Ferrier, Eric E. Grossman, Nathan R. VanArendonk

**Affiliations:** 1 Washington State Department of Natural Resources, Olympia, WA, United States of America; 2 Olympia, WA, United States of America; 3 U.S. Geological Survey, Pacific Coastal and Marine Science Center, Santa Cruz, CA, United States of America; University of Sydney, AUSTRALIA

## Abstract

Kelp forests form an important biogenic habitat that responds to natural and human drivers. Global concerns exist about threats to kelp forests, yet long-term information is limited and research suggests that trends are geographically distinct. We examined distribution of the bull kelp *Nereocystis luetkeana* over 145 years in South Puget Sound (SPS), a semi-protected inner basin in a fjord estuary complex in the northeast Pacific Ocean. We synthesized 48 historical and modern *Nereocystis* surveys and examined presence/absence within 1-km segments along 452 km of shoreline. Compared to the earliest baseline in 1878, *Nereocystis* extent in 2017 decreased 63%, with individual sub-basins showing up to 96% loss. Losses have persisted for decades, across a range of climate conditions. In recent decades, *Nereocystis* predominantly occurred along shorelines with intense currents and mixing, where temperature and nutrient concentrations did not reach thresholds for impacts to *Nereocystis* performance, and high current speeds likely excluded grazers. Losses predominated in areas with elevated temperature, lower nutrient concentrations, and relatively low current velocities. The pattern of long-term losses in SPS contrasts with stability in floating kelp abundance during the last century in an area of the Salish Sea with greater wave exposure and proximity to oceanic conditions. These findings support the hypothesis that kelp beds along wave-sheltered shorelines exhibit greater sensitivity to environmental stressors. Additionally, shorelines with strong currents and deep-water mixing may provide refugia within sheltered systems.

## Introduction

Humans have altered coastal ecosystems for centuries, yet we frequently lack long-term datasets to quantify changes and their outcomes. While the need for long-term reference points was initially identified in the context of global fisheries [1], it is equally important to understand changes in biogenic habitats, as they are sensitive to disturbance, and losses can trigger changes to broader ecosystem structure and services [2, 3].

Kelp forests (order Laminariales) provide biogenic habitat to a wide range of species [4]. Kelp forests are considered ecosystem engineers [5] because they create structural habitat with distinct local conditions by modifying the physical environment, such as light, water flow, sedimentation, and pH [6, 7]. Extremely high productivity rates create habitat and food for local and distant food webs [8, 9]. Because kelp generally requires cold and nutrient-rich water [10, 11], large-scale climate cycles or changes can influence kelp abundance [12–14]. Grazing from herbivores also strongly influences kelp distribution and abundance [15], with changes in herbivory pressure often linked to changes in predator populations [4, 16, 17].

Kelp forest losses across the globe have generated widespread concern and highlighted considerable data gaps. A recent worldwide synthesis found that 38% of kelp forests declined, although one-third of ecoregions were excluded due to insufficient data, and many analyses were confined to less than 10 years [18]. Divergent trends in nearby locations suggest that, in additional to regional climate trends, local factors can dominate kelp dynamics. Broad-scale and local hydrodynamics have been hypothesized to play a role in areas including British Columbia [19], Nova Scotia [11], Maine [17], and western Norway [20], but the role of water motion is difficult to evaluate because it is rarely independent of other factors [10]. In British Columbia, kelp responses to a broad-scale marine heat wave were mediated by fine-scale environmental heterogeneity; wave-exposed habitats remained stable while wave protected sites experienced near complete losses in kelp diversity [19].

Widespread human activities can impact kelp, including development, agriculture, and forestry [5]. In the last decade, kelp declines and shifts from systems dominated by kelp to turf-forming algae have been documented in Asia, Australia, Europe, North America, and South America (reviewed in [21]). Researchers have identified warming [11, 22], eutrophication [20], acidification [23], changes to community structure [17], and sedimentation [24] as contributing factors that often interact. Other known threats include harvest, pathogens, and non-native algal species [25]. As awareness of losses grows along with predictions for future losses associated with climate warming, scientists and managers have identified a pressing need for better baseline information to provide context for past and predicted environmental changes [26, 27] and to determine the extent to which changes are related to human activities.

**Fig 1:**
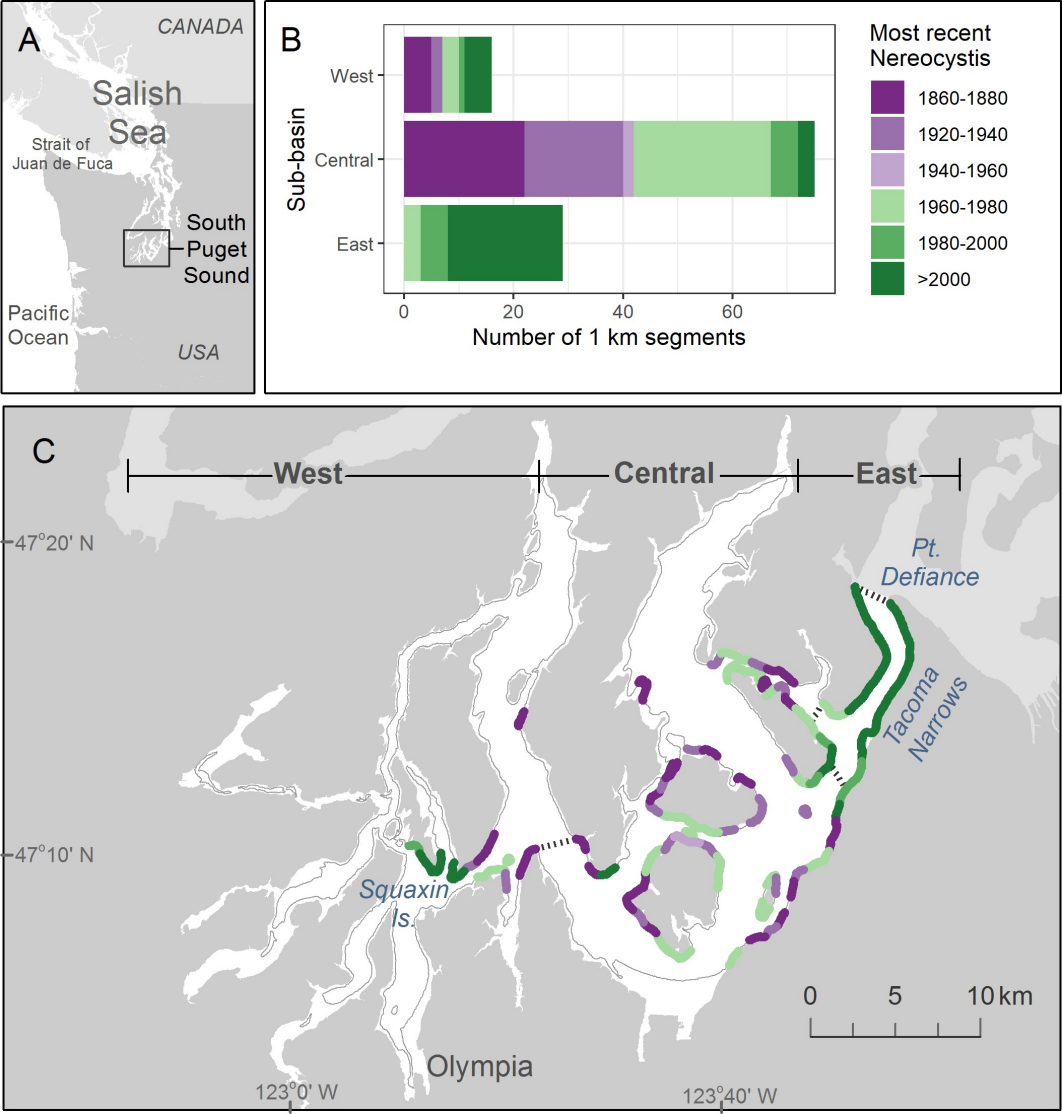
Most recent observation of *Nereocystis* presence along shorelines in South Puget Sound (SPS) between 1873 and 2018. (A) The location of SPS, the southern terminus of the Salish Sea. (B) Bar charts show the most recent year *Nereocystis* was present in 1-km segments within each sub-basin. Years were binned into 20-year increments, with two bins excluded due to lack of data. (C) The -6.1 m bathymetric contour line denotes all shorelines where *Nereocystis* occurrence was assessed, classified by the most recent observation of presence (same legend as in B). The gray line denotes absence throughout the time period. The general location of three sub-basins (West, Central and East) is defined at the top of the map, and dotted gray lines on the map identify precise boundaries. Map image based on publicly available data from the Washington State Department of Natural Resources.

The Salish Sea is an extensive fjord estuary complex in the northeast Pacific Ocean that spans the United States and Canada (Fig 1) and the Puget Trough/Georgia Basin ecoregion [28]. The Salish Sea supports 22 species of kelp [29] with the greatest abundance and diversity found along the western Strait of Juan de Fuca [14, 29]. While kelp is less abundant in the inner basins of the fjord complex, bull kelp *Nereocystis luetkeana* and the understory kelp *Saccharina latissima* are common [30–32] where appropriate habitat conditions exist, such as coarse substrates in the shallow subtidal zone for holdfast attachment. The Salish Sea has been excluded from studies of kelp trends due to data gaps [5, 18]. In adjacent ecoregions along the open Pacific Ocean coast of Washington, Oregon, and British Columbia, kelp abundance was highly variable on interannual timescales, yet stable over longer time periods [14, 18, 33, 34]. An exception to this general pattern was seen in the eastern extreme of the Strait of Juan de Fuca, where abundance decreased along the shorelines at the entrance to the Salish Sea over the past 100 years [14]. This finding suggests that kelp dynamics may differ within the Salish Sea.

South Puget Sound (SPS) represents a geographic and oceanographic end point to the Salish Sea, and it provides an extreme contrast to the adjacent ecoregion for considering long-term trends in kelp. It is the most distant basin from oceanic influence and it naturally experiences lower flushing rates and longer water residence times, which make it particularly sensitive to extreme climate conditions and water quality degradation [35, 36]. Human activities impact water conditions in SPS through point and non-point pollution sources associated with regional development in the SPS watershed, as well as in nearby Central Puget Sound [37].

In this study, we focus on Bull kelp *Nereocystis luetkeana* (hereafter *Nereocystis*), the sole species of kelp in the Salish Sea that forms a floating surface canopy. *Nereocystis* occurs from the Aleutian Islands, AK, to Point Conception, CA [38], and is one of the most common species of canopy-forming kelps [39], occurring in a wide range of habitats from fully wave-exposed to moderately wave-sheltered shorelines [40]. *Nereocystis* is a ruderal or opportunistic species that thrives following disturbances, exhibits high fecundity and exhibits higher interannual variability than perennial kelp species with which it often co-occurs [10, 14, 34, 39]. In the absence of disturbance, *Nereocystis* can be replaced by perennial kelp species [16]. The macroscopic sporophyte phase of *Nereocystis* is primarily an annual, with a holdfast that attaches to coarse substrates, a long stipe connected to a terminal buoyant bulb, and blades that proliferate on the water surface. Like other species of kelp, the sporophyte has an obligatory alternate phase, a microscopic gametophyte, whose ecology is poorly understood and may be vulnerable to different environmental factors [10, 41, 42].

In California and Oregon, satellite imagery has been used to retrospectively examine multi-decadal trends in kelp canopies [13, 34]. However, this technique can be unreliable in portions of the northeast Pacific Ocean where narrow, low-density *Nereocystis* beds hug the shoreline, strong currents and extreme tides limit acceptable imagery to narrow time windows, and clouds commonly obscure imagery [43–45]. While long-term monitoring data are lacking, diverse data sources have noted the occurrence of *Nereocystis* in SPS since European exploration began in the mid-1800s. Historically, kelp surface canopies were charted as an aid to navigation [46]. More recently, surface canopies have been surveyed for environmental monitoring and resource management [39]. In addition to these canopy-focused studies, intertidal and dive-based subtidal ecological studies (i.e., [32]) have quantified density and other metrics for *Nereocystis* over more limited spatial scales.

Here, we synthesized diverse historical and modern data sources in order to understand spatiotemporal patterns in *Nereocystis* in the SPS and linkages to contemporary observations of climate, water temperature, nutrient concentration, and wave/current energy. We hypothesized that: 1) *Nereocystis* extent contracted in SPS relative to its historical extent, 2) shifts in *Nereocystis* extent were not explained by short-term climate conditions, and 3) *Nereocystis* losses occurred in areas that experienced elevated water temperature, low nutrient concentration, and low wave and current energy in recent decades. Like other syntheses of diverse historical datasets (i.e., [20, 47]), the primary purpose of our assessment is to describe changes over time. We do not have the temporal resolution to fully assess inter-annual variability in kelp abundance, but we can identify broad patterns and relate them to climate conditions at the time of the observations. While we cannot draw conclusions about the causes of observed changes, we can place results in the context of regional data and draw inferences about some likely stressors. Improved understanding of the historical extent of kelp and patterns of change could support further research into stressors and target restoration and conservation actions. It could also increase our understanding of dynamics in the organisms that rely on these habitats.

## Methods

### Study system

The South Puget Sound (SPS) study area is a 425 km^2^ water body located at the southern terminus of Puget Sound, within the Salish Sea fjord estuary complex, (Fig 1, approximate location 47.1791093 N, 122.7858146 W). SPS is connected to the northeast Pacific Ocean through a network of deep water basins and shallow sills. Circulation is dominated by tidal currents, superimposed on a two-layered estuarine circulation pattern with a deep inflow of oceanic waters and a surface outflow driven by freshwater input from rivers [36, 48, 49]. SPS has a relatively shallow euphotic zone. Marine nutrient concentrations are naturally high and can be drawn down by strong spring and summer algae blooms with nutrient limitation and eutrophic conditions evident in some areas [50]. Individual basins such as SPS are strongly affected by terrestrial conditions, and longer water residence times make them sensitive to nutrient addition and other types of water quality degradation [36, 51]. SPS is the shallowest major basin (mean depth 37 m) in Puget Sound [48] and has the highest diurnal tidal range, 4.4 m in Olympia [52]. Tidal currents and wave action primarily drive water flow and vertical mixing. The most intense currents and daily mixing in SPS occur at the Tacoma Narrows, a narrow 1.5 km channel with a shallow sill (45 m) that connects SPS to the rest of Puget Sound. The upper water column is well mixed in the central channels of SPS where kelp has been recorded [35].

SPS has complex shorelines composed of islands, passages, and shallow inlets. Wave exposure is low, ranging from semi-protected to very protected in the regional shoreline classification dataset called the ShoreZone Inventory [53]. Due to the area’s glacial origin, gravel, sand, and mixed fine substrates from eroded glacial till and outwash predominate in the intertidal and shallow subtidal zones [30, 54]. Mixed coarse substrates are found along shorelines with strong currents and relatively long fetch. Tide flats of mud or sand predominate at the heads of the inlets and other shallow embayments [30, 48].

Native people have inhabited the region for more than 12,000 years [55]. Limited European settlement began in SPS in the 1820s, and SPS contained the largest population in Puget Sound in 1870 [56]. After 1870, population growth in the cities of Seattle and Tacoma outpaced SPS. The Puget Sound region is now extensively urbanized, with a regional population of more than 4 million in 2019 [57]. A number of human activities have impacted natural systems since European settlement began, with lumber production dominating economic activity. Other important economies in SPS have included fishing, agriculture, and aquaculture. In recent decades, impacts associated with urbanization predominate. Development has brought extensive nearshore habitat loss and degradation [58], with attendant water quality issues pertaining to anthropogenic nutrient loads and contaminants from urban, industrial, and agricultural runoff.

### Kelp survey data synthesis

We compiled 48 individual data sources that noted the presence or absence of *Nereocystis* in SPS, including peer-reviewed publications, maps, charts, reports, and field surveys [30, 32, 59–103]. The datasets spanned from 1873 to 2018 and were produced for a wide range of purposes, including navigation, harvest, resource management, land use planning, and ecological research. The spatial extent of data sources varied from a single location to the entire study area. Format and level of detail also varied widely, including text descriptions of presence or absence at a location, generalized cartographic symbols, delineations of bed perimeter, and phycological studies, which examined detailed plant metrics such as density and phenology. When multiple versions of a data source existed, we chose the most detailed field survey in preference to the final product, which was often edited for cartographic presentation. For example, we selected the hydrographic sheets [59] and the accompanying descriptive reports used to create the navigation charts, whenever available. For the Coast Pilot, which was updated gradually over time, records were grouped into three time periods (1889, 1926, and 1951) following the methods of Thom and Hallum [104]. In eight cases, original data sources were unrecoverable, so we drew data from a compilation of historical data sets [61, 66–68, 70, 86, 87, 93].

We selected a data synthesis approach to accommodate diverse datasets and also recognize two major known sources of uncertainty that limit the precision of *Nereocystis* canopy observations. First, canopy-forming kelp exhibits high inter-annual variability in extent [14, 105], so a single delineation will generally be a less representative measure of multi-year conditions than for slower growing, longer lived biogenic habitats like coral reefs. Second, tides and currents affect the portion of the canopy that is visible on the water surface over short time periods (hours) in this region [43]. These limitations called for a synthesis approach that generalized *Nereocystis* observations to the most comparable format.

We developed a linear model to represent *Nereocystis* presence along the shoreline because the majority of sources depicted the approximate location of *Nereocystis* rather than precisely delineating the canopy footprint. The linear model captured the common narrow, fringing bed morphology of *Nereocystis* along the SPS shorelines. We selected a -6.1 m (MLLW) bathymetric contour line because it represents a generalized maximum depth of *Nereocystis* beds in SPS and more consistently reflects the linear extent of available *Nereocystis* habitat than intertidal contour lines. A relatively high-quality -6.1 m (MLLW) digital isobath exists, derived from gridded bathymetric data [106].

For all mapped surveys, we transferred information on survey extent and kelp presence from individual data sources to the common bathymetric contour in a geospatial database using ArcMap 10.6.1 [107]. We split the contour line to denote *Nereocystis* presence/absence alongshore, with a minimum mapping length of 3 m. Features were generally one or more orders of magnitude larger than the minimum mapping unit (S1 Text, S1 Fig). Because many sources depicted *Nereocystis* presence, rather than precise extent, we subsequently generalized the linear data by summarizing presence/absence over 1-km segments of shoreline. We systematically divided the isobath into 1-km segments, defining 459 segments along the 452 km study area. Of these, 14 segments (3%) deviated from the 1-km length by more than 15%. Twelve segments measured less than 1 km, all occurred along shorelines and islands where the isobath was not an integer multiple of 1 km. Two offshore shoals exceeded 1 km (1.2 and 1.3 km, respectively).

We recorded 3,352 instances of *Nereocystis* presence/absence between 1873 and 2018 at 1-km segments. At segments where *Nereocystis* occurred at least once, the number of presence/absence observations per segment over the entire time period ranged from 6 to 13, with a median of 8. We employed the entire pool of observations to explore patterns in most recent *Nereocystis* occurrence and overall persistence (defined as the proportion of all observations at each 1-km segment that noted *Nereocystis* presence). We assessed spatiotemporal patterns in *Nereocystis* distribution using seven synoptic snapshots, separated by 4 to 44 years, that each comprehensively surveyed the study area over a limited time period (Table 1). For simplicity, we refer to each synoptic snapshot by the year it was collected or by the year that the majority of segments were surveyed. We limited the comparison of synoptic surveys to the spatial extent of the smallest survey, which excluded eight 1-km segments at the northeastern boundary of the study area. The seven synoptic snapshots included 93% of the presence/absence observations. Finally, we assessed changes from the oldest historical baseline in 1878 to the most recent synoptic snapshot, conducted in 2017. These two surveys were highly detailed and based on extensive field surveys (Table 1). To compare patterns of kelp abundance and distribution over time within sub-areas, we divided the SPS basin into 3 sub-basins (Fig 1), which partitioned the area along a gradual seasonal gradient in water temperature and nutrient concentrations [35, 51].

**Table 1:** Synoptic *Nereocystis* surveys completed in South Puget Sound.

Survey Years	Purpose	Reference Year	Data Source Description	Scale
1873–1879	navigation	1878	Topographic sheets nos. 1327a, 1327b, 1671, 1672, 1674 1528 [59, 60, 62–65]. Surveyed in the field on plane tables [108]. Geo-referenced maps [109] were aggregated into a synoptic snapshot.	1:10,000
1911–1912	harvest	1911	Kelp beds suitable for harvest were identified as part of the west coast-wide Fertilizer Investigations [69]. Beds delineated on final maps were wider than actual bed width denoted on preparatory maps.	1:100,000
1935–1936	navigation	1935	Hydrographic surveys nos. 5931, 6102, 6103, 6104, 6105, 6106, 6107, 6108, 6197, 6198, 6199, 6202, 6203, 6204, 6205 [71–85]. The source data for navigation charts, included field surveys of soundings and aids to navigation. Multiple maps were aggregated into a synoptic snapshot.	1:10,000–1:20,000
1978	habitat	1978	Washington Department of Wildlife field survey maps [92], annotated in pencil on a paper hydrographic chart, source for the Coastal Zone Atlas [110].	1:100,000
1997–1999	habitat	1999	WA State ShoreZone Inventory, based on low tide helicopter-based videography [30, 53]. Classified *Nereocystis* alongshore presence as patchy (<50%) or continuous (>50%) within geomorphically defined linear shoreline units	1:24,000
2013	habitat	2013	GPS-based small boat survey that noted presence of *Nereocystis* as a line feature along the -6.1 m bathymetry line and as a polygon feature for beds of concern.	1:12,000
2017	habitat	2017	GPS-based small boat survey that noted presence of *Nereocystis* as a line feature along the -6.1 m bathymetry line and as a polygon feature for beds of concern [103]. Minimum linear length of 3 m. A 10 m threshold between alongshore plants was applied to classify gaps as ‘absent’. Threshold for *Nereocystis* presence was one bulb.	1:12,000

### Climate conditions

We assessed climate conditions at the time of kelp surveys using the Ensemble Oceanic Niño Index (ENS ONI), a publicly available climate index which spans the study time period and exhibits robust correlations with Multivariate ENSO index, the Extended Multivariate ENSO Index and other indices [111]. We compared the year of each kelp survey to mean ENS ONI during the growing season (March–August) [34]. ENS ONI values during the growing season correlated strongly with the mean of the previous 12 months (r = 0.769, df = 152, p < .001), another predictor employed in kelp and climate comparisons [14].

### Water temperature, salinity and nutrient concentration data

We explored the relation of surface water temperature, salinity, and nutrient concentrations to observed kelp distribution through two data sets: long-term data from mid-channel stations, and a single year from nearshore stations. The datasets allowed us to compare spatial patterns at mid-channel vs nearshore locations and temporal patterns over multiple decades vs one year.

#### Long-term, mid-channel conditions

**Fig 2:**
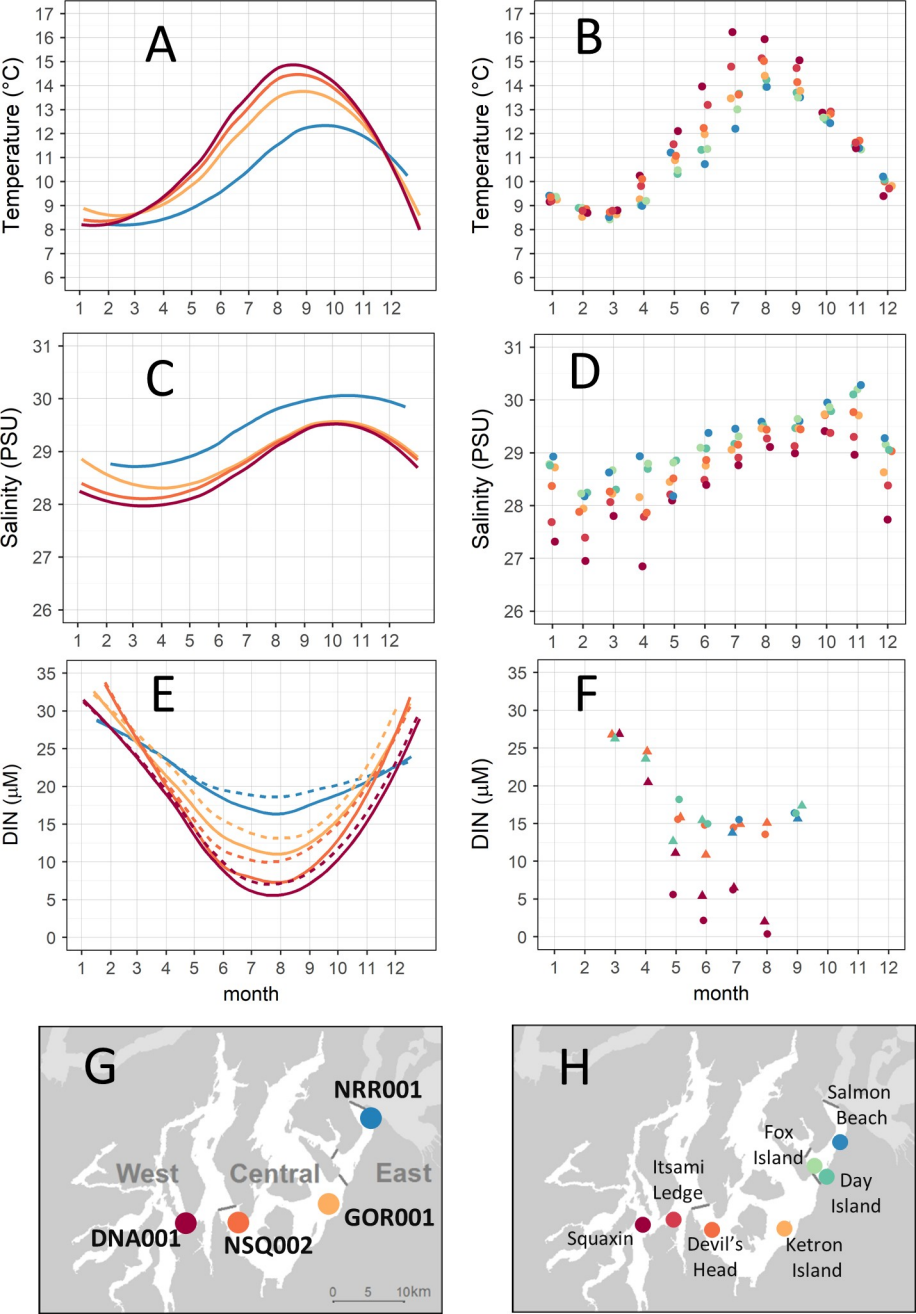
Monthly water characteristics at mid-channel long-term monitoring stations (left) and nearshore stations (right). (A, B) mean water temperature to 5 m depth. (C, D) mean salinity to 5 m depth. (E) DIN concentration at mid-channel stations at depths of 0 m (solid line) and 10 m (dashed line). (F) DIN concentration at nearshore stations at depths of 0.25 m (point) and 4 m (triangle), with data slightly offset horizontally for visibility. Site locations for (G) mid-channel stations and (H) nearshore stations. Mid-channel long-term monitoring station data (left) represent a cubic spline curve fit to mean values from more than 2 decades of sampling for all stations except NRR001 (3 years). Nearshore stations (right) were sampled monthly at -6.1 m (MLLW) between September 2017 and August 2018. Map image based on publicly available data from the Washington State Department of Natural Resources.

We described long-term water properties with publicly available data from four mid-channel water quality stations, sampled monthly by the Washington State Department of Ecology [112]. We restricted our analysis of salinity and temperature to the top five meters of the water column from continuous vertical profiles. We summed nitrate, nitrite and ammonium concentrations to represent dissolved inorganic nitrogen (DIN) at the two shallowest reported depths for nutrients (0 m and 10 m). Monthly values for each parameter at each depth for all available years were reduced to a representative annual pattern using locally estimated scatterplot smoothing (LOESS). More than two decades of monthly data exist at three of the stations (Fig 2): DNA001 (1989–2016), NSQ002 (1996–2016) and GOR001 (1996–2016). Observations were limited to three years at NRR001 (1989–1991).

#### Recent nearshore conditions

We characterized recent nearshore water column properties along an axis from the entrance to SPS at Tacoma Narrows to the most distal documented kelp forests using data collected at seven nearshore sites monthly from September 2017 to August 2018. This axis encompasses a known environmental gradient [112], and sites were placed near historical and recent *Nereocystis* beds (Fig 2). All sites were surveyed on the same day, within two hours of solar noon, during low tide and low current periods. A sampling station was established in the center of each site along the -6.1 m (MLLW) bathymetric contour. A weighted SonTek Castaway®-CTD measured temperature and salinity. We calculated mean salinity and temperature for the top 5 m of the water column. From March to September 2018, field-filtered water samples were also collected to measure DIN concentrations at four sites as time permitted (Squaxin Island, Devil’s Head, Day Island, Salmon Beach, Fig 2). From May to September, samples were taken from each site at 0.25 and 4 m depths in order to assess possible surface water stratification during late-spring and summer. In March and April, only 1 sample was collected at 4 m depth. An acid washed 60 mL syringe with an attached 0.45 μm cellulose acetate filter was filled with water directly from a Van Dorn sampler. A small amount of water was filtered through the syringe to rinse the syringe and syringe filter before rinsing an acid washed 60 mL high density polyethylene bottle with filtrate. The bottle was then filled with filtrate before being placed immediately in a cooler on ice and transported to the Evergreen State College laboratory where they were frozen (-10˚ C) for later transport to the University of Washington’s Marine Chemistry Lab for total dissolved nutrient analysis using spectrophotometric methods.

### Wave and current exposure characterization

We used modeled current velocity and wave heights to explore the relationship between these physical factors and spatiotemporal patterns in *Nereocystis*. We limited these comparisons to segments where *Nereocystis* was observed at least once (n = 120).

We characterized current speed using modeled surface water velocity data from a Salish Sea circulation model [36, 113, 114]. We used data from the surface layer, which represents the top 3% of the water column, from model year 2014. We calculated the annual average of the maximum daily flow velocity (m/s) based on the flow velocities in the x and y directions at each model node. We then summarized velocity with a single value for each 1-km shoreline segment by selecting the value from the closest model grid point. Median distance between model node points and the nearest point on the corresponding shoreline segment was 91 m.

We characterized wave energy using average annual maximum wave height data developed by the Washington Coastal Resilience Project using the numerical wave model SWAN (Simulating WAves Near Shore) [115]. The model generated a hindcast of hourly wave conditions across the Salish Sea over a 60-year period between 1950 and 2010. Modeled values were sampled along the -10 m (NAVD88) bathymetric isobath for SPS and quantified the average annual maximum wave height over the 60-year hindcast. The hindcast utilized the 12-km Weather Research and Forecasting historical reanalysis of the Pacific Northwest [116, 117], which was found to represent the spatial patterns of extreme wind events well but bias wind speeds slightly lower (~1 m/sec) than observed winds over water. Because of this slight bias and because significant wave heights characterize the upper 33% of the wave-height distribution, the wave heights reported here are likely underestimates. We summarized the wave data every 1-km alongshore by selecting the wave model grid point closest to each 1-km segment. Median distance between the model point data and the corresponding shoreline segment was 3.4 m.

### Statistical analyses

All data analyses were performed in ArcGIS version 10.6.1 [107] and R 3.6.0 [118]. Data analysis of kelp extent was based on descriptive statistics of spatial and temporal patterns, the disparate methods used across data sources limited the analyses that could be conducted.

We tested if temperature was different among nearshore water column sites (Fig 2) during summer (June to September) or winter (November to February) using two mixed effects models (one for each season) with a random factor of month (temperature ~ site, random = 1|month), with the R packages “nlme” [119] and “emmeans” [119]. We tested if residuals were normally distributed using qqplots and Shapiro-Wilk tests and visually assessed model output for patterns in normalized residuals.

We tested for differences among sub-basins in current speed and wave height at segments where *Nereocystis* was observed at least once using Welch’s ANOVA to accommodate unequal variance and unequal sample sizes, with the R package “userfriendlyscience” [120]. The wave data were normally distributed, and the current data were log transformed to approximate a normal distribution.

## Results

### *Nereocystis* extent declined and spatial distribution shifted

Based on all available data sources, *Nereocystis* occurred at least once along 26% of the SPS shoreline (120 of 459 1-km shoreline segments) between 1873 and 2018. *Nereocystis* never occurred in the extreme reaches of any inlets (Fig 1). The East sub-basin contained the greatest proportion of recent occurrences; *Nereocystis* occurred at 72% of the segments since 2000 and at all segments since 1960 (Fig 1). In contrast, at the majority of segments in the Central and West sub-basins, the most recent *Nereocystis* occurrence was approximately 4 decades ago (89% and 63% prior to 1980, respectively).

**Fig 3:**
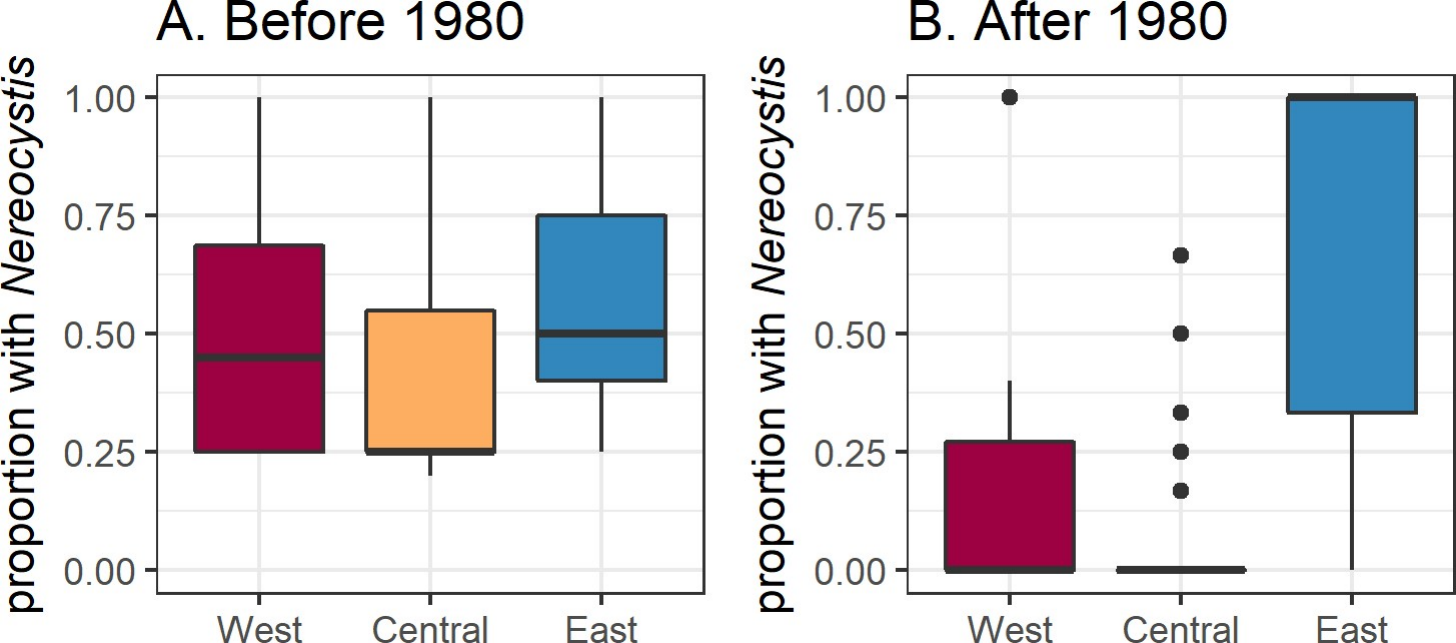
Distribution of *Nereocystis* persistence at 1-km segments (A) before 1980 and (B) after 1980. Persistence was calculated as the proportion of all observations in each segment with *Nereocystis* present within each time period. All 1-km segments where *Nereocystis* occurred at least once in either time period were included (n = 120).

Persistence, measured as the proportion of data sources that noted *Nereocystis* presence within each 1-km segment, ranged from 0.1 to 1, with a median of 0.3 (S2 Fig). There was a marked difference among sub-basins in *Nereocystis* persistence before and after 1980 (Fig 3). Before 1980, persistence was similar in West and East (median values of 0.45 and 0.5, respectively) and lower in Central (median = 0.25). After 1980, median persistence dropped to 0 in the West and Central sub-basins. In the East sub-basin, median persistence increased to 1.0, reflecting widespread, high *Nereocystis* presence. The broad range of persistence values in the East sub-basin after 1980 primarily reflected more observations of *Nereocystis* absence in the southwestern portion of the Tacoma Narrows (especially within sector 26, S2 Fig).

**Fig 4:**
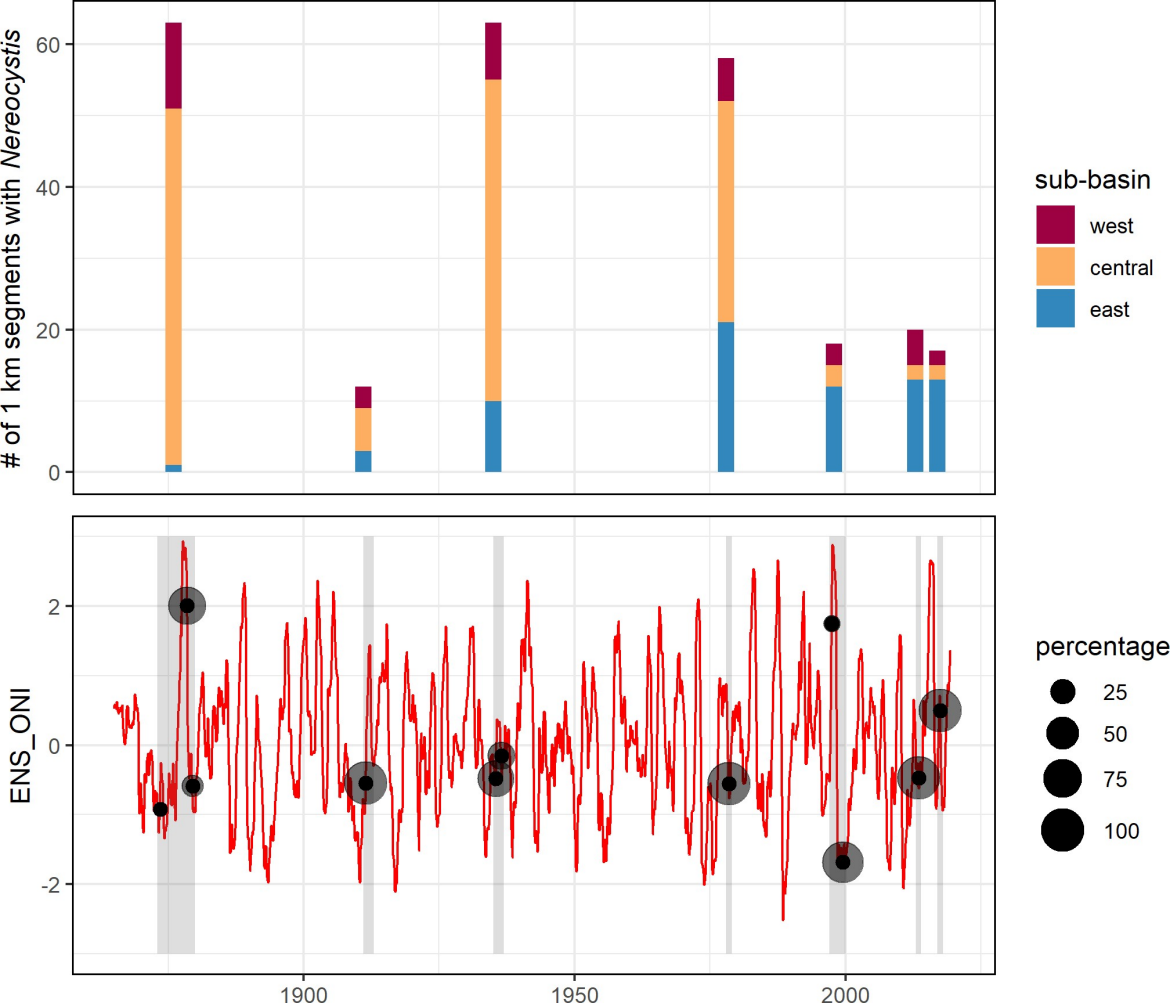
*Nereocystis* extent between 1878 and 2017 in SPS. (A) Number of 1-km segments with *Nereocystis* present, based on seven comprehensive snapshot surveys, summarized over three sub-basins. Recent estimates (1999, 2013 and 2017) are dramatically reduced relative to estimates in 1878, 1935 and 1978. The 1911 estimate could represent a low point in kelp extent, but likely reflects methodological differences in survey methods (commercial beds). (B) Time series of the Ensemble Oceanic Niño Index (ENS ONI) [111]. Gray shading indicate years of synoptic snapshot data collection (some spanned multiple years). Black points identify mean ENS ONI values during the growing season in the year of kelp surveys for synoptic snapshots. Gray circles are scaled to represent the percentage of all segments surveyed for a synoptic snapshot during individual years.

The extent of *Nereocystis* present in synoptic snapshots fell into two distinct groups (Fig 4): approximately 60 1-km segments with *Nereocystis* were identified in 1878, 1935, and 1978 (63, 63, and 58 segments, respectively). In contrast, *Nereocystis* occurred in one-third or fewer segments in 1911, 1999, 2013, and 2017 (12, 18, 20 and 17 segments, respectively). Differences among datasets in total extent likely reflect both changes in kelp distribution and methodological differences among surveys. The 1911 estimate was the lowest, and it starkly contrasts with the high estimates before (1878) and after (1935). The 1911 estimate could have marked a minimum in *Nereocystis* extent; however, the purpose of the survey (to identify beds with harvest potential) could have led to identification of a limited number of large, accessible beds rather than an exhaustive survey (Table 1, S1 Text, S1 Fig, Results section). The three most recent estimates (1999, 2013, and 2017) showed dramatically restricted extent relative to 1878, 1935, and 1978, and similar extent to 1911.

The synoptic snapshots showed a marked shift in the spatial distribution of kelp forests among sub-basins (Fig 4). The Central sub-basin contained the majority of shorelines with *Nereocystis* in 1878, 1911, 1935, and 1978 (79%, 50%, 71% and 53%, respectively). In contrast, the Central basin contained 17% or less of the total extent in the three most recent surveys (17% in 1999, 10% in 2013, and 12% in 2017). The West sub-basin generally contained a smaller proportion of the total shoreline with *Nereocystis* than the Central sub-basin (12–25%); in 2017 they contained equal portions of the total extent (12% each). Decreases in Central and West corresponded to proportional increases in total extent in the East: the proportion in the East ranged from 2–44% during the earliest four surveys, and 65–76% in the three most recent surveys.

Compared to the earliest baseline in 1878, *Nereocystis* extent in 2017 decreased 63% throughout the SPS study area. The most extreme losses occurred in the Central sub-basin (96%), followed by the West sub-basin (83%), while the East sub-basin increased.

### *Nereocystis* data sources represent diverse climate conditions

*Nereocystis* data sources reflect a wide range in climate conditions. El Niño conditions (positive ENS ONI anomalies which are unfavorable for kelp) predominated in the earliest and the most recent synoptic snapshots, while relatively neutral or cool La Niña conditions predominated in the middle decades (Fig 4). High variability was also evident over short time periods. The most unfavorable ENS ONI growing season value (2.0) occurred in 1878 when 72% of the study area was surveyed during an extreme El Niño [121]. The remaining 28% of the segments within that synoptic snapshot were surveyed in cooler climate conditions (ENS ONI was -0.9 and -0.6 during the growing season in 1873 and 1879, respectively). Cooler, favorable climate conditions predominated during 1911 (-0.5), 1935 (-0.5 for 66% of segments and -0.2 during 34% of segments), and 1978 (-0.6). The majority of the 1999 synoptic snapshot (88%) occurred during strong La Niña conditions in 1999 (-1.68), while a minor portion (12%) occurred during a strong El Niño in 1997 (1.8). The penultimate synoptic snapshot captured cool conditions in 2013 (-0.5). Four years later, the final survey in 2017 captured warm conditions (0.5) following a strong El Niño in 2014–2016 and global marine heatwave [122].

### Sea surface temperature and nutrient data show strong gradient

Water temperature, salinity, and nutrient concentration data displayed strong seasonal and spatial patterns that were similar at the long-term mid-channel stations and the nearshore stations sampled in 2017–2018 (Fig 2). For the winter nearshore temperature data, the likelihood ratio test indicated that including site did not improve the final mixed effects model (L = 4.7, df = 1, p = 0.034), indicating that average winter water temperature did not differ among sites. In contrast, nearshore summer water temperatures varied markedly among sites (L = 52.52, df = 1, p < 0.001). Post-hoc tests confirmed that the magnitude of differences in summer temperature increased with geographic distance between sites. Minimum annual temperatures (8°C) occurred during February/March, with less than 1°C difference among all stations. From March to October, the warmest water consistently occurred in the West sub-basin at adjacent locations in Dana Passage (DNA001 mid-channel station) and Squaxin Island (nearshore station), with slightly higher measurements at nearshore stations. The highest overall water temperature recorded at a nearshore station occurred at Squaxin Island in July 2018 (16.2°C). The coolest spring-fall temperatures consistently occurred in the East sub-basin at adjacent locations in the Tacoma Narrows (NRR001 mid-channel station) and Salmon Beach (nearshore station). At Salmon Beach, nearshore temperature peaked at 13.9°C in August 2018. Central sub-basin water temperatures fell midway between the extremes measured at Tacoma Narrows and Dana Passage at all the mid-channel and nearshore stations, with a consistent gradient of values increasing with distance from the Tacoma Narrows. The water column at all nearshore stations was well mixed even in July during peak annual temperatures, with less than 0.5°C range per cast between the surface and 5 m depth [103].

Salinity ranged from 28–30 PSU (long-term curve-fit) at mid-channel stations, with similar values and annual patterns at nearshore stations. Salinity was higher in the summer and late fall, a common pattern in the region associated with seasonal rainfall and freshwater input cycles. Extreme salinity values occurred at the geographic extremes of Tacoma Narrows and Dana Passage. Nearshore salinity ranged from 27.1 PSU in February at Squaxin Island to 30.3 PSU at Salmon Beach in November.

Dissolved inorganic nitrogen (DIN) concentrations at mid-channel stations were high in the winter months at all stations: 25 μM and greater at depths of 0 m and 10 m (Fig 2). Values diverged throughout the spring, with pronounced differences among sites from May to October. Concentrations were slightly higher at depth. A strong spatial gradient emerged, with decreasing concentrations into SPS and the most extreme drawdown of nutrients at Dana Passage, where the long-term mean fell below 10 μM at both 0 m and 10 m depth from June to September. Nearshore DIN concentrations showed a similar pattern for all months with data. Nearshore concentrations were indistinguishable at stations sampled during March 2018, ranging from 26–26 μM at 4 m depth. Concentrations at Squaxin dropped every successive month until August, reaching the lowest concentration measured at any site in August 2018 (0.4 μM at 0.25 m depth). In contrast, nearshore DIN concentrations at the other stations never dropped below 10 μM.

### Recent *Nereocystis* observations predominated in high current areas

**Fig 5:**
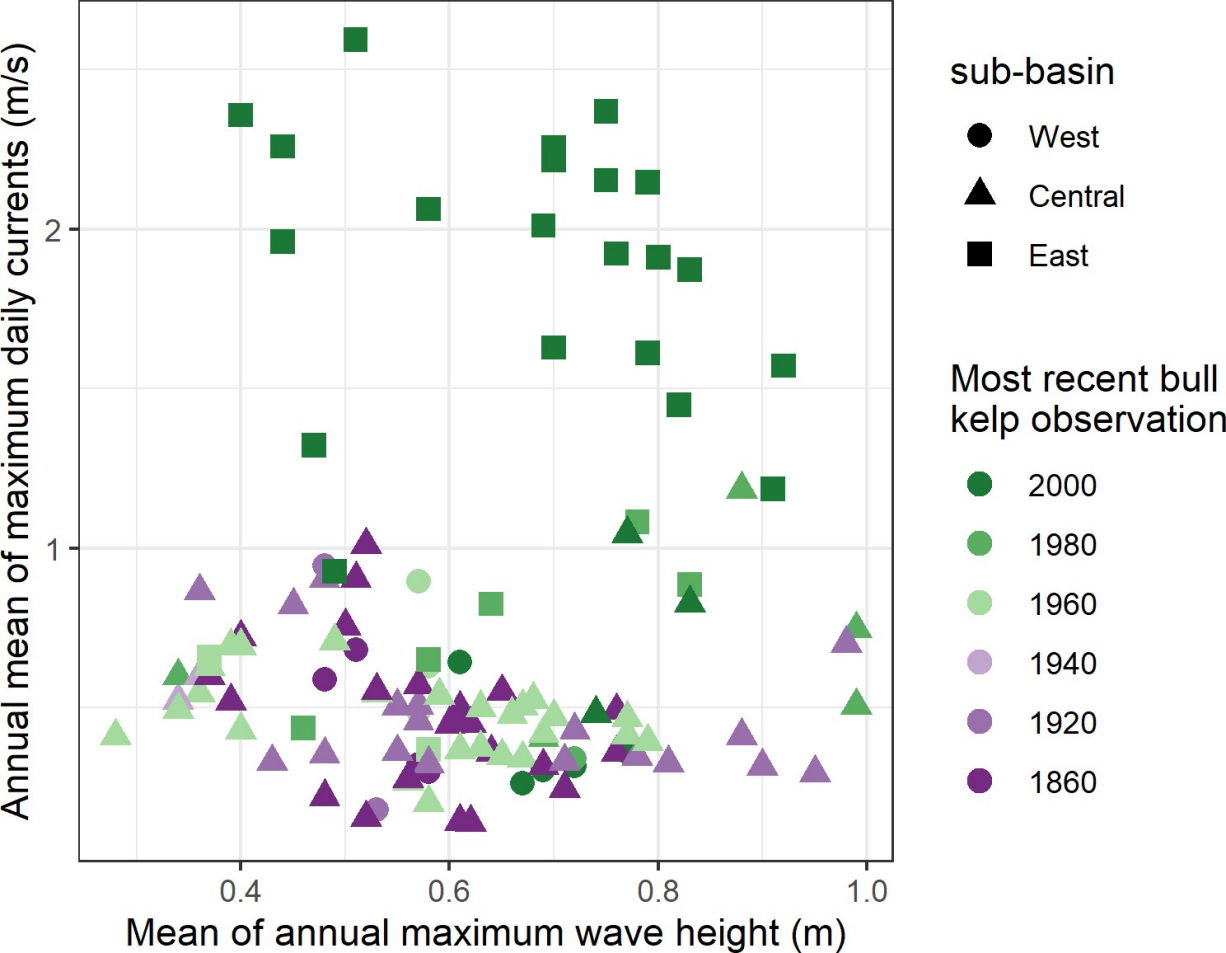
Current and wave exposure at 1-km segments with *Nereocystis*. The annual mean of maximum daily current velocity (y-axis) was derived from a 2014 model run of the Salish Sea Model [110, 111]. Average annual maximum wave height (x-axis) was modeled between 1950 to 2010 by the Washington Coastal Resilience Project [112]. 1-km kelp segments are coded by sub-basin and the most recent year that *Nereocystis* was observed (n = 120).

Average maximum daily current velocities ranged from 0.14 to 2.59 m/s (median 0.52 m/s) at segments where *Nereocystis* was observed at least once during the entire study period (Fig 5). These current velocities differed among sub-basins (Welchs ANOVA test: F(2, 116) = 61.85, p < 0.001). The East sub-basin experienced significantly larger current velocities than the West and Central sub-basins (Games Howell post hoc test, p<0.001), where the median value (1.63 m/s) was approximately 4 times larger than in the West (0.42 m/s) and Central (0.48 m/s) sub-basins. Comparison of historical *Nereocystis* observations with current velocity data showed all of the segments where *Nereocystis* has not been observed since 1980 or earlier experienced current velocities of 1 m/s or less. After 2000, the majority of segments with *Nereocystis* were restricted to shorelines with mean maximum daily currents above 1 m/s, which were predominantly located in the East sub-basin. However, the segments where *Nereocystis* persisted in West and Central sub-basins range in current velocity from 0.31–0.82 m/s.

Average annual maximum wave height ranged from 0.28 m to 0.99 m (median 0.62 m) at segments where kelp was observed at least once during the entire study time period throughout SPS. Wave height did not differ among sub-basins (Welchs ANOVA test: F(2, 116) = 0.7, p = 0.50) but displayed high spatial variability dependent on shoreline orientation. Locations facing south/north recorded higher annual maximum wave heights, indicative of the dominant wind directions in Puget Sound. Median values within the sub-basins ranged from 0.58–0.70 m. No patterns were evident over time in the range of wave heights at segments where *Nereocystis* was observed (Fig 5).

## Discussion

### Major *Nereocystis* losses and shift in distribution

This study established a historical 1878 baseline for *Nereocystis* distribution in SPS, early in the period of European settlement. We described major losses from that baseline in both extent and distribution over 145 years. The most extreme decreases occurred in the Central and West sub-basins; the most recent dataset identified a single location with *Nereocystis* remaining in each of these sub-basins (S2 and S3 Figs). Many of the observed losses in the West and Central sub-basins have persisted for four decades or longer, in a range of climate conditions. In contrast, the East sub-basin appeared stable or increasing.

The observed trend of *Nereocystis* decrease in SPS over 145 years contrasts sharply with findings along the Strait of Juan de Fuca, at the entrance to the Salish Sea. There, kelp forest area generally remained stable over the last century, except along the eastern boundary—the area farthest from oceanic influence and closest to anthropogenic development [14]. This contrasting pattern of adjacent sub-regions experiencing loss and stability has occurred in other locations globally [11, 17, 19, 20].

### Conditions associated with observed patterns in *Nereocystis*

The pattern of kelp contraction over time was apparent across both extreme and neutral ENSO conditions. The longest temporal comparison (1878 and 2017) captured *Nereocystis* extent during two extremely unfavorable El Niño events. More recently, relatively low kelp extent occurred during both favorable conditions (2013) and El Niño conditions following a profound marine heatwave (2017). This finding strongly suggests that the long-term pattern of *Nereocystis* loss is not an artifact of climate conditions at the time of the surveys. While the dataset lacks the temporal resolution for a statistical comparison of kelp and climate indices, it is striking that high/low *Nereocystis* extent did not track favorable/unfavorable climate conditions more strongly. We believe the relatively coarse scale of mapping (presence/absence along 1-km segments), which likely masked differences in abundance that occurred in response to climate conditions. At intensively monitored sites, differences in abundance measurements within 1-km segments were detected between 2013 and 2017 [103].

Climate indices provided a tractable method to broadly compare conditions in this study; however, within the Salish Sea they do not correlate as tightly to temperature, and presumably other oceanographic conditions, as along shorelines on the exposed coast [123–125]. Also, the Salish Sea has experienced higher magnitude increases over recent decades in SST than along the adjacent exposed coastline and global averages [123–125]. One hypothesis, which we lack the data to test, is that *Nereocystis* losses in SPS occurred as a series of contractions in response to discrete climate events and other stressor pulses, with recovery between these events limited by long-term temperature increases, other ongoing stressors, and increasing distance between spore sources. While the historical data in this study lacked sufficient temporal resolution for us to quantify the relationship to climate indices, two notable climate events corresponded to observed *Nereocystis* declines. The 1978 synoptic snapshot provided the most recent measure of high *Nereocystis* extent, and it was followed by a large decline which coincided with a shift from a cold to a warm regime defined in Pacific Decadal Oscillation (PDO) data [126]. Following the 1978 snapshot, small scale surveys showed a gradual disappearance from many segments in the subsequent two decades, culminating in a substantially reduced magnitude in the 1999 synoptic snapshot (S2 Fig). Similarly, smaller declines occurred between 2013 and 2017, coinciding with a period of warm sea surface temperature in the northeast Pacific Ocean known as ‘the Blob’ [127]. Since then, small scale surveys have recorded disappearance at additional segments (S2 and S3 Figs). Marine heatwaves doubled globally between 1982 and 2016 and are projected to become more frequent and extreme [128].

While temperature and nutrient concentration data were not available for the majority of the study period (1870s-1980s), long-term data from recent decades characterized general conditions and short-term data demonstrated similar patterns mid-channel and near shore. Elevated temperatures and low nutrient concentrations occurred in tandem in SPS, a pattern seen in upwelling systems along the exposed coast and in other areas within the Salish Sea [12, 129–133]. In SPS, summer temperatures were shown to be considerably higher and nutrient concentrations were lower compared to the exposed coast and Strait of Juan de Fuca where upwelling and ocean mixing drive water column properties [134]. Many studies have shown that kelp is sensitive to high temperatures and low nitrogen. An 18-year study concluded that temperature increases from a thermal outfall were associated with the virtual disappearance of *Nereocystis* [135]. In British Columbia, elevated temperatures have been associated with lower abundance of *Nereocystis* [105] and other kelp species [19]. In SPS, *Nereocystis* appeared stable in the East sub-basin, where all temperature measurements in this study remained below the proposed range (14.5–16°C) for a physiological thermal threshold for *Nereocystis* [34] and also below the 14.0°C threshold identified in southern California for compromised *Macrocystis* performance related to nutrient concentrations [136]. In contrast, recent summer temperatures at nearshore stations and long-term mid-channel means in the West and Central sub-basins consistently fell within or exceeded the proposed threshold for *Nereocystis* (and always exceeded the lower *Macrocystis* threshold). Additional sampling within the Squaxin Island kelp forest documented even higher temperatures than at the nearshore stations, ranging from 17 to 20°C [103]. These temperature maxima approached or exceeded thresholds for decreased resilience of sporophytes and increased zoospore mortality [42, 137, 138].

Nitrogen requirements for *Nereocystis* are not precisely defined, yet data suggest that low summer concentrations in recent decades may be affecting *Nereocystis* performance in portions of SPS. The authors observed that the *Nereocystis* blades at Squaxin Island were thin, short and shredded. In field studies of nitrogen fertilization, concentrations of 10 μM were associated with thicker blade tissues and a lower rate of blade erosion in *Macrocystis* sporophytes [139]. Laboratory studies showed increased performance in microscopic stages of *Nereocystis* associated with DIN increases from 1 to 15 μM [42]. Nitrogen requirements are likely greater during periods of rapid growth or elevated temperatures [140]. In Puget Sound, anthropogenic inputs are a major local source of DIN [141]. However, worldwide research predicts that elevated anthropogenic nutrient loads damage kelp performance by stimulating growth of phytoplankton [36] and nuisance algae, and introducing particulates and other pollutants and contaminants [21, 142]. While long-term trends are not well understood, anthropogenic DIN inputs in Puget Sound have altered dissolved oxygen levels and algal biomass [36], and the nutrient balance appears to have shifted in recent decades with potential impacts to species composition and material cycling [143, 144].

Exceptions exist to the general pattern of *Nereocystis* losses in sub-basins with higher temperatures and lower nutrients. The innermost bed at Squaxin Island remained highly persistent despite the poorest measured environmental conditions. This exception demonstrates that *Nereocystis* can persist in elevated temperature and low nutrient conditions. The authors believe that the site’s long fetch from prevailing southern winds and extensive appropriate shallow subtidal habitat contribute to *Nereocystis* persistence. However, even there, intensive surveys found that total bed area and maximum depth decreased between 2013 and 2018 [103].

Many studies have demonstrated that hydrodynamic exposure to waves and currents influences kelp dynamics directly and indirectly. Along exposed coastlines, physical disturbance through extreme wave events can drive kelp mortality [13]. Low water motion can limit the capacity of kelp to acquire nutrients and eliminate waste products [5, 145]. In relatively sheltered environments in other regions, wave exposure metrics were positively correlated with greater kelp performance and negatively correlated with elevated temperature [19]. SPS has a relatively protected wave environment, with short-period waves (<5 sec) that have significantly less energy than long-period ocean swell in other habitats where *Nereocystis* occurs. In SPS, current velocity is the primary source of daily water turbulence, and strong tidal currents lead to lower temperatures and higher nitrogen levels through mixing, especially at the Tacoma Narrows [146]. These factors could explain the observed pattern of *Nereocystis* losses in low current areas versus persistence in recent years in intermediate and high current areas. Areas of intense mixing may constitute kelp refugia from physical stressors, and low current areas may exacerbate the negative effects of stressors. The extent that waves further mix the water column to mitigate or compound other stressors to kelp in areas of low tidally driven circulation remains uncertain but likely important given observed variability in wind speeds and directions spatially and at interannual to decadal time scales.

Currents can also mediate biotic stressors. Sea urchins, the most studied kelp grazers, were observed to be absent or rare in SPS. The small snail *Lacuna vincta* has been shown to play an important role in mortality to *Nereocystis* in hydrodynamically quiescent habitats in The Salish Sea [40]. While *Lacuna* snails were not commonly observed in SPS in 2017 and 2018, kelp crabs (*Pugettia producta*) were abundant on the blades, bulbs and stipes in the *Nereocystis* forests that were not subjected to regular, intense currents. Kelp crabs preferentially consume fresh *Nereocystis* in Puget Sound, and laboratory and field experiments suggest that they may play an important role in mediating the growth and survival of *Nereocystis* in the Salish Sea [147, 148].

Many other factors that are known to drive kelp abundance, and are outside the scope of this study, likely also played a role in the observed changes in *Nereocystis* distribution. Estuarine circulation, winds and waves exhibit interannual variability, and perhaps long-term trends, that could be particularly important to habitat conditions in areas with low tidal currents. Human activities—especially logging and coastal development—have increased sediment [149–152], nutrient [142], and pollutant loads to coastal ecosystems [141]. These factors are associated with the global ‘flattening of kelp forests,’ through altering competitive interactions with turf algae [21]. In SPS, widespread deforestation began in the mid-1850s, and extensive clear-cut logging and river channelization radically altered sediment delivery to nearshore marine areas and sediment dynamics [153]. Fishing pressure often alters grazer populations by decreasing top-down controls from predation [4, 16, 17]. In SPS, rockfish [154] and other groundfish [155], salmonids [156], and forage fish [157] populations have been dramatically reduced relative to historical levels. These species occupy middle to high trophic positions, directly and indirectly influencing populations of kelp grazers [17, 158]. Alterations to trophic dynamics can also facilitate competition between *Nereocystis* and other macroalgae. In the absence of disturbance, perennial algae can exclude annual kelp species such as *Nereocystis* [16]. At sites where *Nereocystis* persisted or was lost in SPS, hard surfaces in the shallow subtidal were generally colonized by algae or invertebrates. The prostrate kelp *Saccharina latissima* and the invasive perennial alga *Sargassum muticum*, which can exclude *Nereocystis* [25], were observed at many historical and current *Nereocystis* sites in SPS. Compounding the effects of these diverse stressors, sporophyte mortality may impact basin wide bed connectivity as most spores settle within a few meters of the parent sporophyte [159].

## Conclusion

This study synthesized diverse data sources to fill a gap in understanding of long-term trends in kelp. We identified substantial *Nereocystis* losses over 145 years in South Puget Sound (SPS), the southernmost basin of the Salish Sea. The shift in distribution and extent was consistent over a range of climate conditions and has persisted for decades, which strongly suggests it was not due to short-term, inter-annual variability.

When analyzed with environmental data from recent decades, patterns of *Nereocystis* persistence and loss in SPS provide some insights into potential environmental drivers. As expected based on the literature, *Nereocystis* extent generally decreased along shorelines that experienced higher temperatures and lower nutrient concentrations in recent decades, suggesting that these physical factors played a role in losses. Additionally, in recent decades, *Nereocystis* predominantly occurred along shorelines with intense currents and mixing, where temperature and nutrient concentrations did not reach thresholds for impacts to *Nereocystis* performance and under conditions that can exclude grazers. These patterns suggest that in areas sheltered from waves, intense currents and deep-water mixing may provide refugia from physical and biological stressors.

The contrast between floating kelp losses in SPS and stability along the wave-exposed coastline supports the hypothesis that, in the northeast Pacific Ocean, kelp beds along wave-sheltered shorelines exhibit higher sensitivity to environmental stressors [19]. In addition to factors considered here, human development likely played a role in *Nereocystis* losses through altering physical characteristics and trophic relationships. The ubiquity of wave-sheltered shores [19], and potentially distinct kelp trends in these habitats, suggests that existing regional kelp assessments [18, 160] are incomplete for the northeast Pacific Ocean. These results underscore the importance of assessing kelp across a wide range of physical conditions when assessing long-term trends, causes of change and possible management responses.

## Supporting information

S1 File Data used in this study. Excel spreadsheet with tabular data used in analyses and geographic coordinates identifying the center point of each 1 km shoreline segment.
(XLSX)

S1 Text Comparison of synoptic survey methods and results. (PDF)

S1 Fig Distribution of the length of individual *Nereocystis* features in comprehensive snapshot surveys. The distribution of kelp bed feature length in six comprehensive surveys, ranging from a median of less than 0.1 km (2017) to 1.5 km (1911). Differences in length are likely to be related to both survey resolution and actual length of kelp features.
(TIF)

S2 Fig Persistence of *Nereocystis* at segments and sectors (groups of segments). (A) Map shows persistence at segments, calculated as the proportion of all observations with *Nereocystis* present. Segments where *Nereocystis* never occurred are not shown. The maps also identify sectors (groups of adjacent segments aggregated into stretches of shoreline <10 km in length), with numeric identifiers for each sector and black tick marks delineating boundaries. Gray line denotes shorelines where *Nereocystis* was never recorded. (B) Sectors are identified by reference number listed on map and geographic name. Chart summarizes all observations as presence/absence at the scale of sector by survey year. Color denotes presence (blue) or absence (gray). Shapes represent dataset type. Map image based on publicly available data from the Washington State Department of Natural Resources.
(TIF)

S3 Fig Most recent *Nereocystis* distribution in SPS. The -6.1 m bathymetric contour line is divided into 1-km segments, showing shorelines where *Nereocystis* was present during the most recent survey in 2017 or 2018 (pink), present in at least one previous survey but not in the most recent survey (blue), and never recorded (gray). Map image based on publicly available data from the Washington State Department of Natural Resources.
(TIF)
