# Supplementary material for: Long-term changes in kelp forests in an inner basin of the Salish Sea
Source: PLoS One. 2021 Feb 17;16(2):e0229703. doi: 10.1371/journal.pone.0229703 (PMC7888675; doi:10.1371/journal.pone.0229703)
Supplement: S1 Text — (PDF) [file pone.0229703.s002.pdf]

S1 Text. Comparison of Synoptic Survey Methods

Each of the synoptic surveys employed distinct methods (Table 1). We explored the potential effects of methodological differences by comparing the degree of detail among surveys and considering each survey's purpose and approach. For simplicity, we refer to the surveys by reference year (Table 1).

The 1878 surveys classified a relatively high number of *Nereocystis* features (n=43), the median feature length was 0.6 km (S1 Fig). The 1878 survey was known to be based on extensive field observations because the surveyors delineated detailed topographic maps in the field on plane tables [1], it was also comparatively high resolution (1:10,000 scale).

The 1911 survey had the lowest total number of *Nereocystis* features (n=8), the longest median feature length (1.3 km), and the longest minimum feature (S1 Fig). The smaller number of features and larger size suggest that the data were generalized relative to other datasets and did not capture small kelp beds. Additionally, the map scale (1:100,000) was less detailed than most surveys. Overall, we concur with Thom and Hallum [2] who concluded that the 1911 survey likely targeted larger beds that were appropriate for harvest, and beds that were too small to be commercially viable would have been ignored. Evidence supporting this theory is a small bed near Lincoln Park, Seattle, which the surveyor excluded from his harvest map yet recorded in another study [3]. Another possible factor driving the low total number of beds could have been logistical transportation constraints in the early 1900s. Unlike topographic and hydrographic surveys, project documentation suggests that a single investigator (Dr Rigg) completed all surveys throughout Puget Sound over two years. A single surveyor would have needed to limit surveys to a subset of the total area, or to less than ideal tide and current conditions.

The 1935 survey had comparatively high resolution (1:10,000 to 1:20,000) and survey detail (Table 1, S1 Fig). It included more *Nereocystis* features (n=77) than all synoptic surveys except 2017, and feature

lengths were relatively small (median = 0.2 km). The high density of nearshore soundings and detailed delineations recorded on the working maps provide evidence of extensive field data collection.

The 1978 surveys identified a large number of *Nereocystis* features (n=42). While median feature length was comparable to other surveys (0.4 km), many features were substantially longer than other surveys, up to 8 km (S1 Fig). The field methodology suggests that it captured many small *Nereocystis* features that might have been excluded from other surveys: 1) Use of a small plane allowed for a rapid, broad area survey during a narrow window of optimal tide and current conditions; 2) The project goal of habitat delineation likely targeted all beds for identification, regardless of bed size. The recording method of hand-delineation on a 1:100,000 chart with a pencil likely buffered the size of *Nereocystis* beds. Considered together, this information suggests that the 1978 survey had high *Nereocystis* detection capability and exaggerated the spatial extent of features.

The 1999 survey employed the most distinct methodology due to its approach of classifying *Nereocystis* as patchy or continuous within geomorphic units (Table 1). A total of 19 geomorphic units of varying length were classified with *Nereocystis* present. Median feature length (1 km) was longer than all other surveys except 1911 (S1 Fig 1). However, feature length is not directly comparable to the other surveys because it was determined by geomorphic characteristics. The method for transferring the 1999 data to 1 km segments was more likely to inflate *Nereocystis* extent than to under-estimate it because a segment was coded to have *Nereocystis* present if any portion of it overlapped a geomorphic unit where *Nereocystis* was noted as either patchy or continuous.

The 2013 survey was relatively detailed, with 37 kelp features and median feature length of 0.2 km (S1 Fig). The survey methods supported a relatively high resolution and high detection rate of *Nereocystis*: 1) it employed narrow tidal elevation and current speed windows; 2) the survey platform - a small boat

meandering in the shallow subtidal - is more likely to detect *Nereocystis* than remote platforms, especially in areas with narrow, low density beds

The 2017 survey was the most detailed kelp survey, containing the most segments (n=81) and smallest segment lengths (median < 0.1 km). The survey methods supported a relatively high resolution and high detection rate of *Nereocystis*: 1) it employed narrow tidal elevation and current speed windows; 2) the survey platform - a small boat meandering in the shallow subtidal - is more likely to detect *Nereocystis* than remote platforms, especially in areas with narrow, low density beds; 3) single bulbs were recorded.

The highly detailed phycological surveys [4-6] confirmed the *Nereocystis* presence/absence observations in the synoptic snapshots.

References

1. Shalowitz A. Shore and sea boundaries. Washington, DC: US Department of Commerce US Coast and Geodetic Survey; 1964. Available from: https://nauticalcharts.noaa.gov/about/docs/history-of-coast-survey/shore-and-sea-boundaries-volume-two.pdf.
2. Thom RM, 1991. LH, editors. Historical changes in the distribution of tidal marshes, eelgrass meadows and kelp forests in Puget Sound. Puget Sound Research '91; 1991.
3. Rigg GB. Seasonal development of bladder kelp. Publ Puget Sound Biological Station. 1917; 1: 309-18.
4. Hodgson LM, Waaland JR. Seasonal variation in the subtidal macroalgae of Fox Island, Puget Sound Washington. Syesis. 1979; 12: 107-12.
5. Maxell BA, Miller KA. Demographic studies of the annual kelps Nereocystis luetkana and Costaria costata (Laminariales, Phaeophyta) in Puget Sound, Washington. Botanica Marina. 1996; 39(3): 479-89.
6. Phillips R. Shallow subtidals surveys by scuba and snorkel at sites in Puget Sound for Seattle Pacific College and University of Washington. Seattle, WA:1962-63. p. 146. Available from: http://https://www.dnr.wa.gov/publications/aqr_nrsh_phillips_dive_log_1962_1963.pdf?fm90e8.
