# Supplementary figures and images for: Long-term changes in kelp forests in an inner basin of the Salish Sea
Source: PLoS One. 2021 Feb 17;16(2):e0229703. doi: 10.1371/journal.pone.0229703 (PMC7888675; doi:10.1371/journal.pone.0229703)

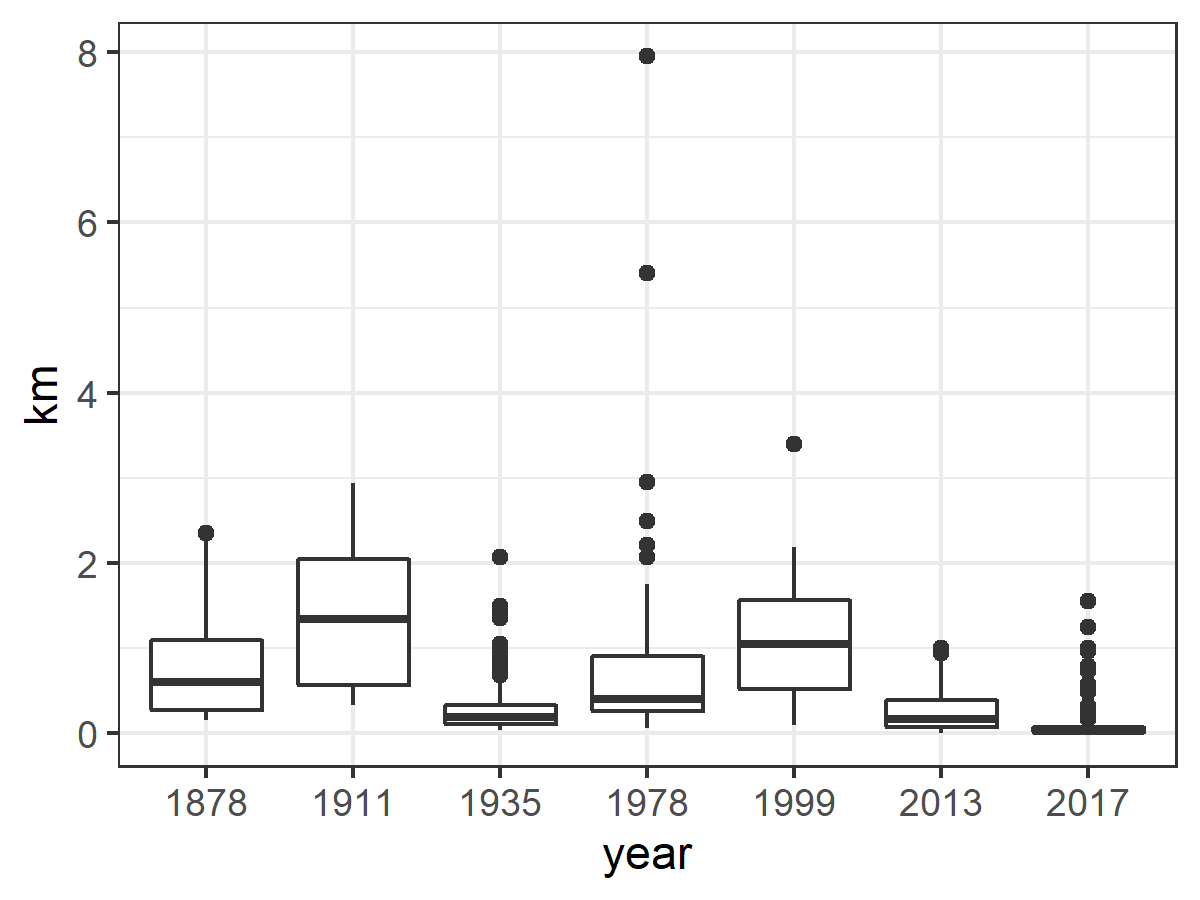

Supplement: S1 Fig — The distribution of kelp bed feature length in six comprehensive surveys, ranging from a median of less than 0.1 km (2017) to 1.5 km (1911). Differences in length are likely to be related to both survey resolution and actual length of kelp features. (TIF) [file pone.0229703.s003.tif]

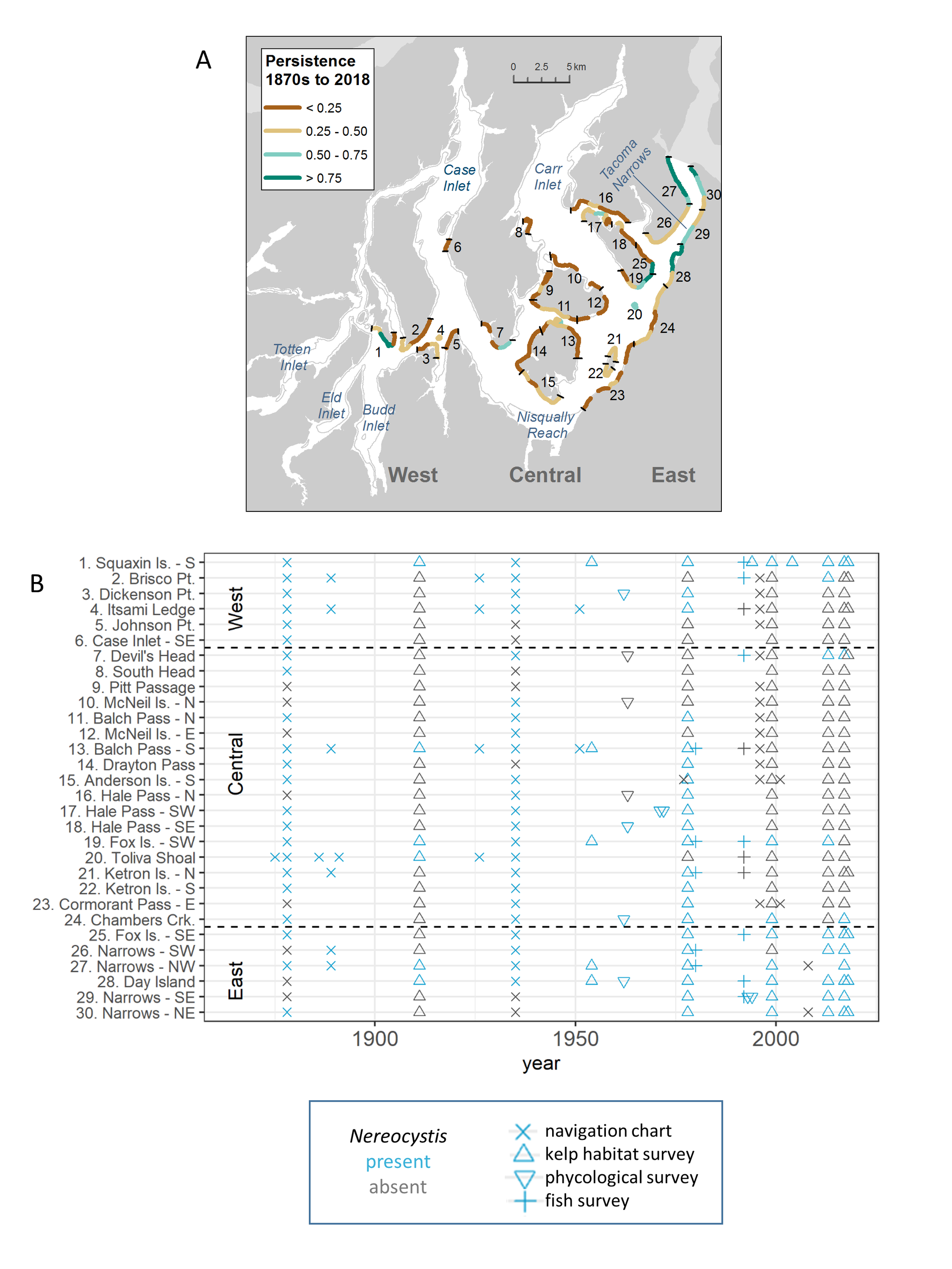

Supplement: S2 Fig — (A) Map shows persistence at segments, calculated as the proportion of all observations with Nereocystis present. Segments where Nereocystis never occurred are not shown. The maps also identify sectors (groups of adjacent segments aggregated into stretches of shoreline <10 km in length), with numeric identifiers for each sector and black tick marks delineating boundaries. Gray line denotes shorelines where Nereocystis was never recorded. (B) Sectors are identified by reference number listed on map and geographic name. Chart summarizes all observations as presence/absence at the scale of sector by survey year. Color denotes presence (blue) or absence (gray). Shapes represent dataset type. Map image based on publicly available data from the Washington State Department of Natural Resources. (TIF) [file pone.0229703.s004.tif]

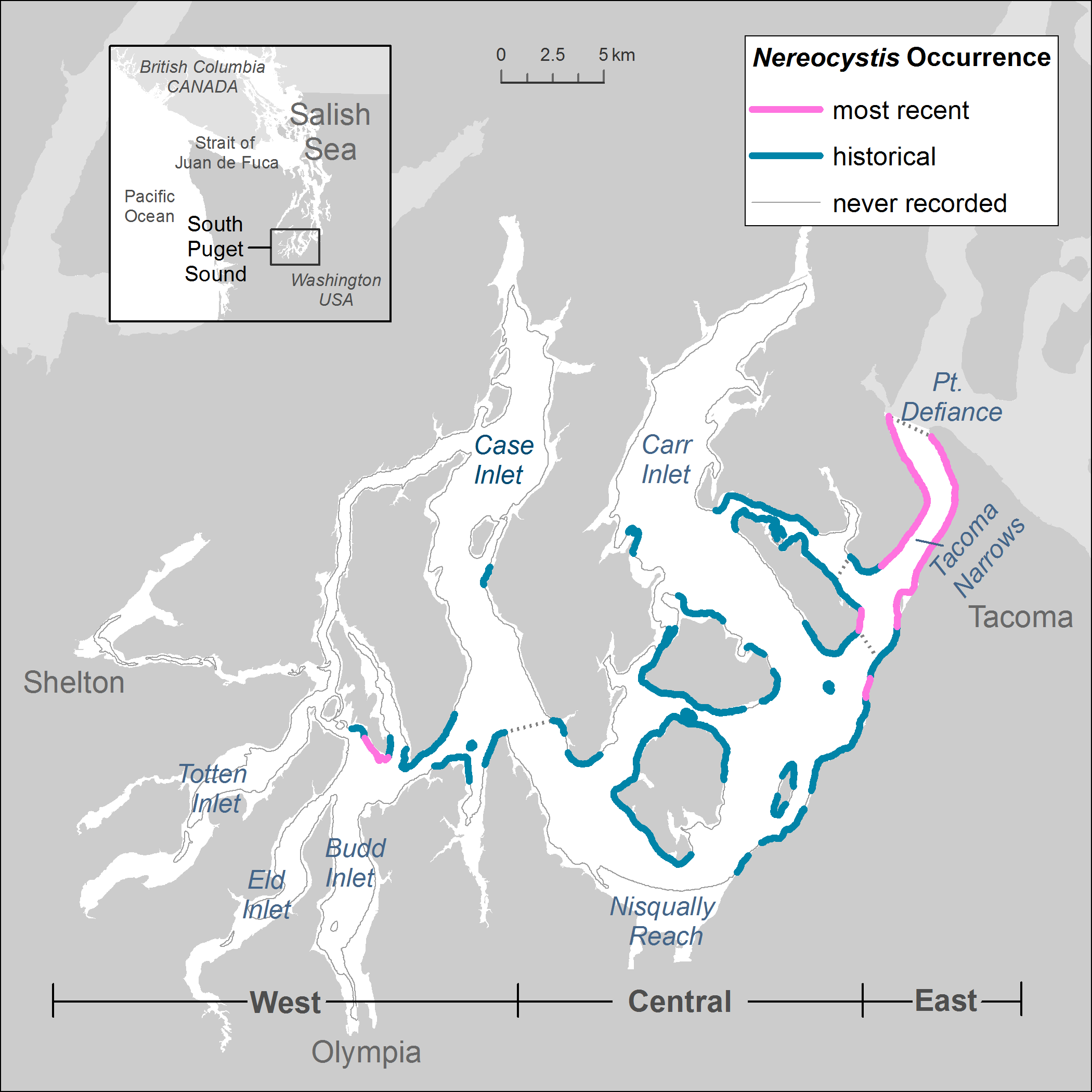

Supplement: S3 Fig — The -6.1 m bathymetric contour line is divided into 1-km segments, showing shorelines where Nereocystis was present during the most recent survey in 2017 or 2018 (pink), present in at least one previous survey but not in the most recent survey (blue), and never recorded (gray). Map image based on publicly available data from the Washington State Department of Natural Resources. (TIF) [file pone.0229703.s005.tif]
